# Pancreatic Cancer Cell-Conditioned, Human-Derived Primary Myotubes Display Increased Leucine Turnover, Increased Lipid Accumulation, and Reduced Glucose Uptake

**DOI:** 10.3390/metabo12111095

**Published:** 2022-11-10

**Authors:** Solveig A. Krapf, Jenny Lund, Awais Ur Rehman Saqib, Hege G. Bakke, Arild C. Rustan, G. Hege Thoresen, Eili T. Kase

**Affiliations:** 1 Section for Pharmacology and Pharmaceutical Biosciences, Department of Pharmacy, University of Oslo, NO-0316 Oslo, Norway; 2 Department of Immunology, Institute of Clinical Medicine, University of Oslo, NO-0318 Oslo, Norway

**Keywords:** cachexia, cross talk, energy metabolism, protein metabolism

## Abstract

Metabolic alterations occurring in cancer cells have been seen to also occur in other tissues than cancerous tissue. For instance, cachexia, peripheral insulin resistance, or both are commonly seen in patients with cancer. We explored differences in substrate use in myotubes conditioned with the medium from a pancreatic cancer cell line, PANC-1, or primary human pancreatic cells, hPECs. Protein turnover was assessed using scintillation proximity assay, glucose and oleic acid handling were analyzed by substrate oxidation assay. We performed qPCR to study gene expression and immunoblotting and proteomic analyses to study protein expression. PANC-1-conditioned myotubes had an imbalance in protein turnover with decreased accumulation, increased decay, and decreased *MYH2* gene expression. Glucose uptake decreased despite increased insulin-stimulated Akt phosphorylation. Fatty acid uptake increased, whereas fatty acid oxidation was unchanged, leading to accumulation of intracellular lipids (TAG) in PANC-1-conditioned myotubes. Secretome analyses revealed increased release of growth factors and growth factor receptor from PANC-1 cells, potentially affecting muscle cell metabolism. Myotubes exposed to pancreatic cancer cell medium displayed altered energy metabolism with increased protein/leucine turnover and lipid accumulation, while glucose uptake and oxidation reduced. This indicates production and release of substances from pancreatic cancer cells affecting skeletal muscle.

## 1. Introduction

Cancer causes great morbidity and mortality and is a major public health concern worldwide. Pancreatic cancer, with pancreatic ductal adenocarcinoma (PDAC) being the most common type, is one of the cancers with the poorest prognosis with a 5-year survival rate of 5% [1].

Metabolic alterations are well-known occurrences with cancer. Cancer cells have higher uptake of glucose and produce more lactic acid, even in the presence of oxygen, compared to normal cells (the Warburg effect) [2]. Alterations in fatty acid metabolism in cancer cells are also increasingly more recognized [2]. Metabolic changes have, however, also been seen to occur in other tissues than the cancer tissue itself.

Skeletal muscle is one of the main regulators of glucose and lipids in the human body and a reservoir of amino acids, with a profound impact on whole-body homeostasis. Cancer cachexia is a syndrome causing weight loss and muscle wasting and is especially seen in striated muscle [3]. Cachexia is caused by factors secreted from (or other types of interactions with) the tumors, including cytokines, such as IL-1, IL-6, TNFα, IGF-1, and myostatin [4]. These interactions leads to an increased basal metabolic rate and energy expenditure, as well as loss of skeletal muscle due to an imbalance in protein synthesis and degradation [4]. Cachexia is found to occur in more than half of all patients with cancer, with an even higher incidence in pancreatic cancer, and is responsible for more than 20% of cancer-related deaths [4]. Furthermore, mitochondrial dysfunction is often seen in cancer-cachexic muscle [5], associated with the accumulation of lipid intermediates and potential insulin resistance in skeletal muscle [6].

Additionally, glucose intolerance is more frequently seen in patients with pancreatic cancer. However, rather than being a risk factor for developing cancer, it is believed to be caused by the presence of the pancreatic tumor itself, as surgical removal of the cancer significantly decreases insulin resistance [7]. Insulin resistance in PDAC is manifested by impaired glucose transport, glucose oxidation, and glycogen synthesis [7]. Dysregulation of lipid metabolism also plays a key role in cancer cells’ success. Cellular homeostasis changes by alterations in fatty acid uptake, synthesis, and hydrolysis [8] to support the rapid proliferation, survival, migration, invasion, and metastasis of cancer cells [8]. Dysregulation of lipid metabolism in pancreatic cancer cells can also be seen in the light of peripheral insulin resistance often occurring in the skeletal muscle of these patients [7] and its role in the pathogenesis of cachexia [8]. For many patients with pancreatic cancer, both cachexia and glucose intolerance are present at the time of diagnosis [9].

Previously, we compared non-malignant primary human pancreatic epithelial cells (hPECs) with a pancreatic cancer cell line (PANC-1) [10]. PANC-1 cells displayed a more glycolytic phenotype, though having higher lactic acid production and being less efficient at metabolizing fatty acids compared to hPECs. These changes were reflected in gene expression and protein secretion. Comparing energy metabolism in human myotubes conditioned with media from hPECs (chPEC exposed), PANC-1 (cPANC-1 exposed), and non-conditioned cells, we found reduced oleic acid oxidation in cPANC-1-exposed compared to non-conditioned myotubes [10]. There is evidence that pancreatic cancer has a great impact on muscle tissue already early in disease development; thus, increasing our knowledge of this interaction could possibly improve the treatment of pancreatic cancer. Therefore, we wanted to further investigate the effects of cPANC-1 and chPECs on energy metabolism in primary human myotubes and to study the possible differences in glucose, lipid, and protein metabolism between the two conditioned myotube cultures.

## 2. Materials and Methods

### 2.1. Materials

PANC-1 cells (ATCC^®^ CRL-1469) were purchased from the American Type Culture Collection (Manassas, VA, USA). Human primary pancreatic epithelial cells (hPECs), human epithelial cell basal medium, and the epithelial cell medium supplement kit were purchased from Cell Biologics (Pelo Biotech, Chicago, IL, USA), and 6-well, 12-well, and 96-well Corning^®^ CellBIND^®^ tissue culture plates were from Corning (Schiphol-Rijk, the Netherlands). Nunc^TM^ Cell Culture Treated Flasks with Filter Caps, Nunc^TM^ 96-MicroWell^TM^ plates, Dulbecco’s Modified Eagle’s Medium (DMEM) high glucose with HEPES and L-glutamine, DMEM-Glutmax^TM^ low glucose with sodium pyruvate, DPBS (without Mg^2+^ and Ca^2+^), FBS, human epidermal growth factor (hEGF), trypsin-EDTA, penicillin-streptomycin (10,000 IE/mL), amphotericin B, Countess^TM^ counting chamber slides, the Pierce^TM^ BCA Protein Assay Kit, TaqMan reverse transcription kit reagents, the High-Capacity cDNA Reverse Transcription Kit, primers for TaqMan PCR, MicroAmp^®^ Optical 96-Well Reaction Plate, MicroAmp^®^ Optical Adhesive Film, and the Power SYBR^®^ Green PCR Master Mix were from Thermo Fisher Scientific (Waltham, MA, USA). Insulin (Actrapid^®^ Penfill^®^ 100 IE/mL) was from Novo Nordisk (Bagsvaerd, Denmark). Trypan blue 0.4% solution, DMSO, gentamicin, dexamethasone, L-glutamine, BSA (essentially fatty acid free), L-carnitine, D-glucose, oleic acid (18:1, n-9), sodium lactic acid, carbonyl cyanide-*4*-(trifluoromethoxy)-phenylhydrazone (FCCP), and HEPES were from Sigma-Aldrich (St. Louis, MO, USA). QIAshredder and the RNeasy Mini Kit were from QIAGEN (Venlo, The Netherlands). The Bio-Rad Protein Assay Dye Reagent Concentrate was from Bio-Rad (Copenhagen, Denmark). D-[^14^C(U)]glucose (18.5 kBq/mL), L-[^14^C(U)]leucine (37 kBq/mL), and [1-^14^C]oleic acid (18.5 kBq) were from PerkinElmer NEN^®^ (Boston, MA, USA). Ultima Gold™, 96-well Isoplate^®^, Unifilter^®^-96 GF/B, and TopSeal^®^-A transparent film were also from PerkinElmer (Shelton, CT, USA). Glucose/Lactate Hemolyzing Solution was purchased from EKF Diagnostics (Cardiff, UK). Antibodies against total and phosphorylated Akt at serine 473 (#9272 and #9271, respectively) and total and phosphorylated AMP-activated protein kinase (AMPK) at threonine 172 (#2531 and #2532, respectively) were from Cell Signaling Technology® (Beverly, MA, USA).

### 2.2. Ethical Approval

Skeletal muscle biopsies were obtained after written informed consent and approval from the Regional Committees for Medical and Health Research Ethics (REK) North, Tromsø, Norway (ref. no. 2011/882). The study adhered to the Code of Ethics of the World Medical Association (Declaration of Helsinki).

### 2.3. Cell Culturing

#### 2.3.1. PANC-1 Cells

PANC-1 cells were cultured in DMEM high glucose (25 mmol/L) supplemented with 10% FBS (PANC-1 medium) according to the manufacturer’s instructions. Cells were kept in 75 cm^2^ Nunc™ flasks before being cultured for experiments and then kept in a humidified in a 5% CO_2_ atmosphere at 37 °C, and the medium was changed every 2–3 days.

#### 2.3.2. hPECs

hPECs were cultured in epithelial cell medium with supplements according to the manufacturer’s instructions (hPEC medium). Cells were kept in 75 cm^2^ Nunc™ flasks before being cultured for experiments and then kept in a humidified 5% CO_2_ atmosphere at 37 °C, and the medium was changed every 2–3 days. Experiments were performed on cells in passages 6–8.

#### 2.3.3. Skeletal Muscle Cells

Biopsies were obtained from the *musculus vastus lateralis* of healthy male donors after obtaining written informed consent and ethical approval. To establish multinucleated human myotubes, satellite cells were first proliferated as myoblasts, as previously described [10], in DMEM-Glutamax^TM^ (5.5 mmol/L glucose) medium supplemented with 10% FBS, 10 ng/mL of hEGF, 0.39 µg/mL of dexamethasone, and 0.05% BSA. At approximately 80% confluence, the medium was changed to DMEM-Glutamax™ (5.5 mmol/L glucose) supplemented with 2% FBS and 25 pmol/L of insulin to initiate differentiation into multinucleated myotubes for 7 days, in a humidified 5% CO_2_ atmosphere at 37 °C, and the medium was changed every 2–3 days. Experiments were performed on cells in passages 3–4.

### 2.4. PANC-1- and hPEC-Conditioned Myotubes

Due to different proliferation rates, an unequal number of PANC-1 cells and hPECs were seeded to achieve an approximately equal cell number at the time of harvesting. PANC-1 cells (~1 × 10^6^ cells/flask) and hPECs (~2 × 10^6^ cells/flask) were cultured in respective media in 75 cm^2^ flasks for 24 h. The cells were washed, and both cell types were cultured in DMEM high glucose (PANC-1 medium without FBS). After 48 h, the media were collected and spun down at 1000 rpm/4 °C/15 min, and the supernatants were frozen at −80 °C. Glucose and lactate levels were measured in the collected media, as described [10], and the glucose concentration was adjusted to be the same in both media (approximately 22 mmol/L). Human skeletal muscle cells were cultured and differentiated in cell culture plates. In the last 4 days of differentiation, the cells were conditioned with the medium collected from hPECs (chPEC) or PANC-1 cells (cPANC-1). The conditioned medium was added in a ratio of 50:50 with DMEM-Glutamax™ (5.5 mmol/L glucose) supplemented with 2% FBS and 25 pmol/L of insulin.

### 2.5. Scintillation Proximity Assay (SPA)

Myotubes were cultured in a 96-well ScintiPlate. After differentiation and conditioning, the myotubes were exposed to [^14^C] leucine (37 kBq/mL, 0.8 mmol/L), followed by measurements of protein (leucine) accumulated in the cells by scintillation proximity assay (SPA) [11]. [^14^C] leucine was monitored over the next 24 h (measurements at 0, 2, 4, 6, 8, and 24 h) using a 2450 MicroBeta^2^ scintillation counter (PerkinElmer). Thereafter, the medium was changed to DMEM with 0.8 mmol/L of leucine, 10 mmol/L of HEPES, 0.5% BSA, and 0.1 mmol/L of glucose, and the decay of [^14^C] leucine was monitored over 6 h (measurements at 0, 2, 4, and 6 h). The amount of radioactivity in the cells was related to the total cell protein content measured according to Bradford [12].

### 2.6. Substrate Oxidation Assay

Skeletal muscle cells were cultured in 96-well CellBIND^®^ microplates. After differentiation and conditioning, D-[^14^C(U)] glucose (18.5 kBq/mL, 200 µmol/L) or [1-^14^C] oleic acid (18.5 kBq/mL, 100 µmol/L) was added during 4 h of CO_2_ trapping [11]. CO_2_ production was measured in the presence or absence of 1 µmol/L of FCCP or 0.5 µmol/L of rotenone in combination with 2.5 µmol/L of antimycin A or 1 µmol/L of oligomycin. CO_2_ production and cell-associated radioactivity (CA) were assessed using a 2450 MicroBeta^2^ scintillation counter (PerkinElmer). The amount of radioactivity in the cells was related to the total cell protein content measured according to Bradford [12]. The sum of ^14^CO_2_ and CA was considered as the total substrate uptake.

### 2.7. Thin-Layer Chromatography and Measurement of Acid-Soluble Metabolites (ASM)

Skeletal muscle cells were cultured in 12-well CellBIND^®^ microplates. After differentiation and conditioning, the myotubes were incubated with [1-^14^C] oleic acid (18.5 kBq/mL, 100 µmol/L) for 4 h. Thereafter, the cells were washed twice with PBS and harvested with 250 µL of 0.1% SDS per well. Cellular lipids were analyzed by extraction of the homogenized cell fraction, separation of lipids by thin-layer chromatography, and quantification by liquid scintillation [13]. A non-polar solvent mixture of hexane:ether:acetic acid (65:35:1) was used to separate the lipids. The amount of neutral lipids was related to the total cell protein concentration determined by Pierce protein assay.

Measurement of acid-soluble metabolites (ASM), reflecting incomplete fatty acid oxidation (β-oxidation) and mainly consisting of tricarboxylic acid cycle metabolites, was performed, as described and modified by Bakke et al. [14]. In short, 100 μL of the radiolabeled medium was transferred to an Eppendorf tube and precipitated with 300 μL of cold HClO_4_ (1 mol/L) and 30 μL of BSA (6%). Thereafter, the tube was centrifuged at 10,000 rpm/10 min/4 °C before 200 μL of the supernatant was measured by liquid scintillation of a Packard Tri-Carb 1900 TR (PerkinElmer, Boston, MA, USA).

### 2.8. Glycogen Synthesis

Differentiated and conditioned cells, cultured in 6-well CellBIND^®^ plates, were starved for 1.5 h in glucose-free DMEM before being exposed to serum-free DMEM (5.5 mmol/L glucose) supplemented with D-[^14^C(U)] glucose (18.5 kBq/mL, 0.67 mmol/L), in the absence or presence of 20 nmol/L or 100 nmol/L of insulin for 3 h. The cells were washed twice with PBS and harvested in 1 mol/L of KOH. Protein content was determined by Pierce protein assay before 20 mg/mL of glycogen and more KOH (final concentration 4 mol/L) were added to the samples. The incorporation of D-[^14^C(U)] glucose into glycogen was measured [15].

### 2.9. Immunoblotting

Myotubes were cultured in 25 cm^2^ flasks and harvested in Laemmli buffer (0.5 mol/L of Tris-HCl, 10% SDS, 20% glycerol, 10% β-mercaptoethanol, and 5% bromophenol blue). Proteins were electrophoretically separated on 4–20% Mini-Protean TGX™ gels with Tris/glycine buffer (pH 8.3), followed by blotting to nitrocellulose membranes and incubation with antibodies. For total Akt and Akt phosphorylation, immunoreactive bands were visualized with enhanced chemiluminescence (Chemidoc XRS, Bio-Rad, Copenhagen, Denmark). and quantified using Image Lab software (version 6.0.1). The level of Akt phosphorylated at serine 473 was normalized for an internal control (HEK cell-line sample) and the total Akt level on the same gel. Immunoreactive bands of total AMPK and AMPK phosphorylation were visualized using Image Studio Lite (version 5.2). The level of AMPK phosphorylated at threonine 172 was normalized for an internal control (HEK cell-line sample) and the total AMPK level on the same gel.

### 2.10. RNA Isolation and Analysis of Gene Expression by qPCR

Skeletal muscle cells were cultured in 6-well CellBIND^®^ plates. After differentiation and conditioning, total RNA was isolated using the QIAGEN RNeasy Mini Kit and reverse-transcribed on a PerkinElmer 2720 Thermal Cycler (25 °C/10 min, 37 °C/80 min, and 85 °C/5 min) using a High-Capacity cDNA Reverse Transcription Kit and TaqMan reverse transcription kit reagents. Primers (Supplementary Table S1) were designed using Primer Express^®^ (Thermo Fisher Scientific, Waltham, WA, USA). qPCR was performed using the StepOnePlus Real-Time PCR system (Thermo Fisher Scientific, Waltham, WA, USA). Target genes were quantified in duplicate, carried out in a 25 µL reaction volume. All assays were run for 44 cycles (95 °C/15 s followed by 60 °C/60 s). Two housekeeping genes were examined, glyceraldehyde 3-phosphate dehydrogenase (*GAPDH*) and ribosomal protein lateral stalk subunit P0 (*RPLP0*). Gene expression levels were comparable regardless of the choice of the housekeeping gene; data shown are presented relative to *RPLP0.* Forward and reverse primers were used at a concentration of 30 µmol/L.

### 2.11. Proteomics

Re-analysis of proteomic data from a previous analysis [10] was performed. Mass spectrometry proteomics data were deposited to the ProteomeXchange Consortium via the PRIDE partner repository (dataset identifier PXD017613). STRING software (version 11.5) was applied to identify functional protein association networks using annotated key words: “growth factors”, “growth factor binding proteins”, and “cytokines”.

### 2.12. Presentation of Data and Statistics

All values are reported as means ± SEM. The value *n* represents the number of individual experiments, each with at least duplicate observations. Statistical analyses were performed using GraphPad Prism (version 8.3.0) for Windows or SPSS Statistics (version 28). The unpaired *t*-test was used to evaluate the effects on myotubes conditioned with either hPEC or PANC-1 medium. The paired *t*-test was used to evaluate the effects of treatment on myotubes conditioned with the medium from either of the two cell types. A *p*-value of <0.05 was considered statistically significant.

## 3. Results

### 3.1. Measurement of Leucine Incorporation in Myotubes Indicated Decreased Protein Synthesis, Increased Protein Decay, and Lower mRNA Expression of MYH2 after Being Conditioned with the Medium from Pancreatic Cancer Cells

**Figure 1:**
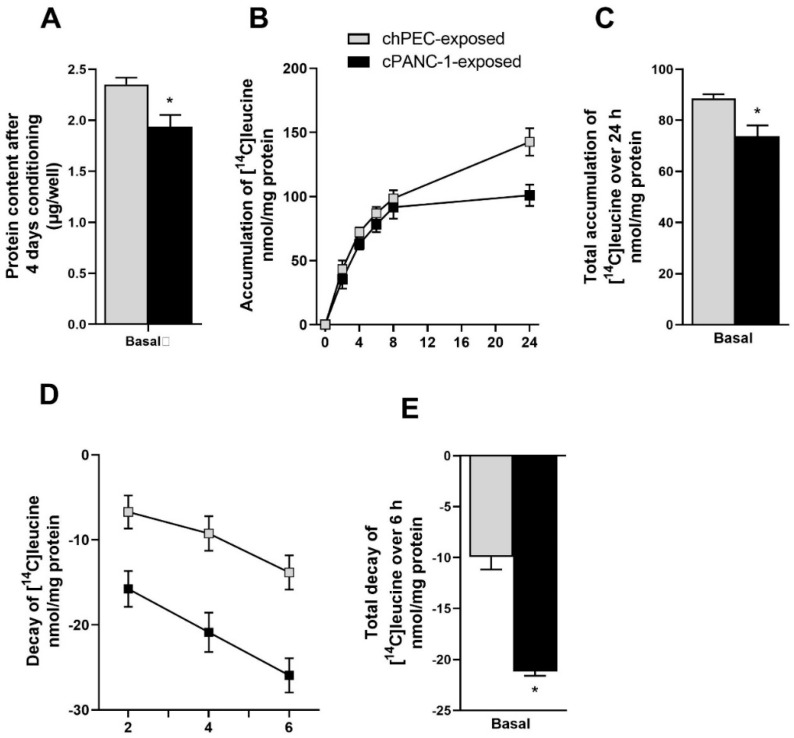
Incorporation and decay of leucine in myotubes after conditioning with medium collected from hPECs or PANC-1 cells. Myotubes were conditioned with the medium from human pancreatic epithelial cells (chPEC exposed) or a human pancreatic cancer cell line (cPANC-1 exposed) during the last 4 days of differentiation. (**A**) Protein content was measured using Bradford protein assay. Muscle cells were incubated with [^14^C] leucine (37 kBq, 0.8 mmol/L), and (**B**) cellular accumulation of leucine was measured over 24 h by scintillation proximity assay (SPA). (**C**) Overall accumulation of [^14^C] leucine was calculated as the area under the curve. (**D**) The medium was then changed to label-free medium, and decay of radioactivity was measured over 6 h by SPA. (**E**) Overall decay over 6 h was calculated as the area under the curve. Data are presented as the mean ± SEM from four individual experiments, each with three replicates (*n* = 12). * Statistically significant for cPANC-1-exposed myotubes versus chPEC-exposed myotubes (*p* ≤ 0.05, SPSS linear mixed-model analysis).

As cancer-induced cachexia leads to increased degradation of protein in skeletal muscle [4], we wanted to examine the protein content in myotubes after conditioning with the medium from hPECs (chPEC exposed) or PANC-1 cells (cPANC-1 exposed). We found that the overall protein content significantly decreased after 4 days of conditioning with cPANC-1 and chPEC media (Figure 1A). To further explore the difference seen in the protein content, we traced the cellular accumulation of [^14^C] leucine as a measure of protein synthesis in myotubes and thereafter evaluated the decay of radioactivity from the cells. The accumulation of leucine over 24 h was significantly less for cPANC-1-exposed myotubes compared to chPEC-exposed cells (Figure 1B,C). Further, the decay of radioactivity measured over 6 h increased for cPANC-1-exposed myotubes compared to chPEC-exposed cells (Figure 1D,E).

**Figure 2:**
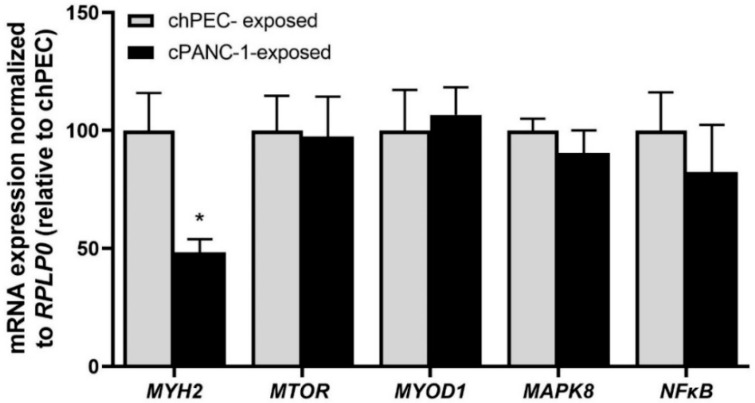
Expression of genes related to protein turnover in myotubes after conditioning with medium collected from hPECs or PANC-1 cells. Myotubes were conditioned with the medium from human pancreatic epithelial cells (chPEC exposed) or a human pancreatic cancer cell line (cPANC-1 exposed) during the last 4 days of differentiation. mRNA was isolated, and the expression of myosin heavy chain 2 (*MYH2*), mechanistic target of rapamycin (*MTOR*), myoblast determination protein 1 (*MYOD1*), mitogen-activated protein kinase 8 (MAPK8), and nuclear factor kappa B (*NFκB*) was assessed. The expression of each gene was normalized for the expression of the housekeeping gene ribosomal protein lateral stalk subunit P0 (*RPLP0*). Data are presented as the mean ± SEM from two individual experiments, with three replicates each (*n* = 6) relative to expression in chPEC-exposed cells. * Statistically significant in cPANC-1-exposed myotubes versus chPEC-exposed myotubes (*p* ≤ 0.05, unpaired *t*-test).

The mRNA expression of selected genes important for protein synthesis and cachexia was also measured. The mRNA expression of ATP hydrolyzing motor protein myosin heavy chain 2 (*MYH2*) significantly reduced in cPANC-1-exposed myotubes compared to chPEC-exposed cells, while no changes were found for the serine-threonine kinase mechanistic target of rapamycin (*MTOR*), mitogen-activated protein kinase 8 (*MAPK8)*, the myogenic regulatory factor myoblast determination protein 1 (*MYOD1*), or the immune response regulator nuclear factor kappa B (*NFκB*); see Figure 2.

### 3.2. Myotubes Conditioned with PANC-1 Culture Medium Had Lower Glucose Uptake and Oxidation Compared to hPEC-Conditioned Myotubes

**Figure 3:**
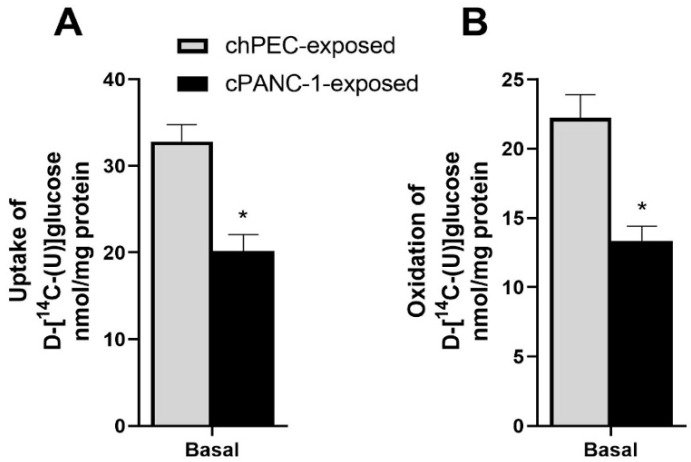
Impact of conditioning myotubes with medium from hPECs or PANC-1 cells on glucose metabolism. In the last 4 days of differentiation, myotubes were conditioned with the medium from human pancreatic epithelial cells (chPEC exposed) or a human pancreatic cancer cell line (cPANC-1 exposed). Thereafter, myotubes were incubated with 200 µmol/L of D-[^14^C(U)]glucose (18.5 kBq/mL) for 4 h before glucose uptake and oxidation were measured. (**A**) Uptake was calculated as captured CO_2_ + cell-associated (CA) [^14^C] glucose. (**B**) Complete oxidation was measured as trapped CO_2_. Data are presented as the mean ± SEM from three individual experiments, each with eight replicates (*n* = 24). * Statistically significant in cPANC-1-exposed myotubes versus chPEC-exposed cells (*p* ≤ 0.05, unpaired *t*-test).

The link between pancreatic cancer and peripheral insulin resistance is not well understood. We wanted to explore whether conditioning skeletal muscle cells with media from hPECs and PANC-1 cells influences glucose metabolism. We found that cPANC-1-exposed myotubes had lower uptake and oxidation of [^14^C] glucose compared to cells exposed to chPEC medium (Figure 3A,B).

### 3.3. Akt Phosphorylation Was Higher under Basal Conditions and When Treated with Insulin in Myotubes Conditioned with PANC-1 and hPEC Culture Media

**Figure 4:**
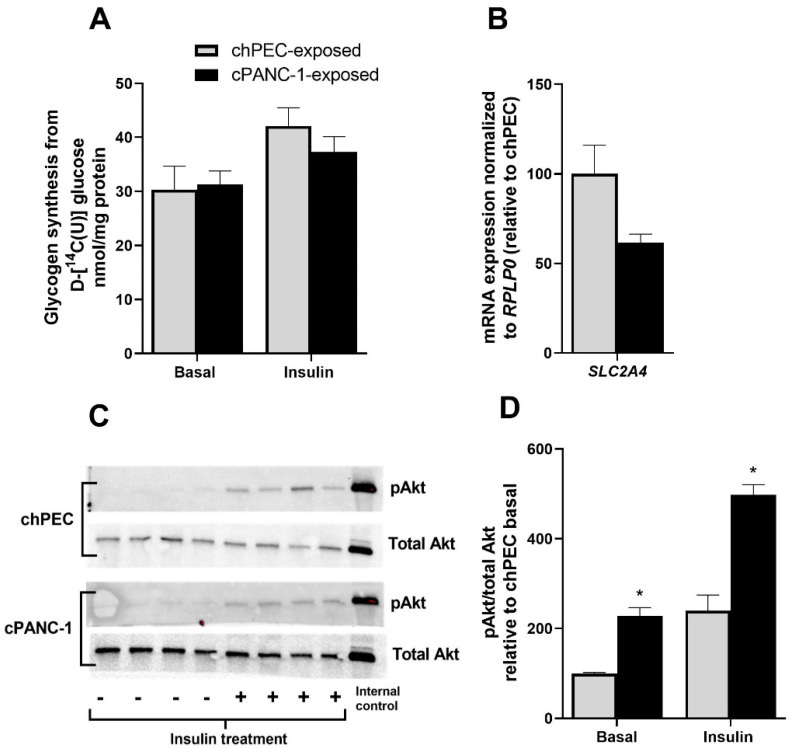
Impact of conditioning myotubes with medium from hPECs or PANC−1 cells on insulin responses. Myotubes were conditioned with the medium from human pancreatic epithelial cells (chPEC exposed) or a human pancreatic cancer cell line (cPANC-1 exposed) during the last 4 days of the differentiation period. (**A**) After starving myotubes for 90 min in a medium containing no glucose, insulin-stimulated glycogen synthesis was measured as the incorporation of D-[^14^C(U)]glucose (18.5 kBq/mL, 0.67 mmol/L) into glycogen in the presence or absence of 100 nmol/L of insulin for 3 h. (**B**) mRNA expression of solute carrier family 2 member 4 (*SLC2A4*) was normalized for the housekeeping gene ribosomal protein lateral stalk subunit P0 (*RPLP0*), and the expression in cPANC-1-exposed myotubes is presented relative to chPEC-exposed cells (*n* = 3–6). (**C**) Immunoblot showing Akt phosphorylated at serine 473 (pAkt) and total Akt and in cells treated with or without 100 nmol/L of insulin for 15 min. (**D**) Quantified immunoblot showing the pAkt/total Akt ratio, adjusted to the internal control (HEK cells) and presented as a percentage of basal chPEC (*n* = 4). Data in (**A**,**B**,**D**) are presented as the mean ± SEM. * Statistically significant in cPANC-1-exposed myotubes versus chPEC-exposed myotubes (*p* ≤ 0.05, unpaired *t*-test).

Insulin-induced glycogen synthesis was not significantly different between chPEC-exposed myotubes and cPANC-1-exposed cells (Figure 4A). There was an insignificant reduction in the mRNA expression of the insulin-dependent glucose transporter *SLC2A4* (*p* = 0.079) in cPANC-1-exposed cells compared to chPEC-exposed myotubes (Figure 4B). To further examine the response of insulin in these cells, we assessed Akt phosphorylation at serine 473 in cells treated with or without insulin for 15 min. Akt phosphorylation was higher in cPANC-1-exposed myotubes compared to chPEC-exposed myotubes, both under basal conditions and after insulin treatment (Figure 4C,D).

### 3.4. Myotubes Exposed to PANC-1-Conditioned Medium Showed Increased Lipid Accumulation and Responded Less to Electron Chain Complex Inhibitors Compared to hPEC-Conditioned Myotubes

**Figure 5:**
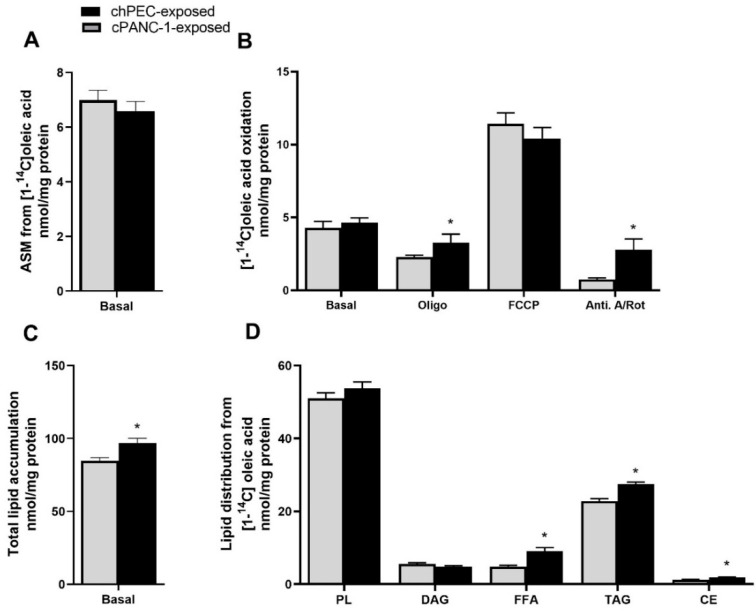
Fatty acid metabolism in PANC-1- and hPEC-conditioned myotubes. In the last 4 days of differentiation, myotubes were conditioned with the medium from hPECs (chPEC exposed) or PANC-1 cells (cPANC-1 exposed). Thereafter, the muscle cells were incubated with [1-^14^C]oleic acid (18.5 kBq, 100 µmol/L) in the presence or absence of 1 µmol/L of oligomycin (electron transport chain (ETC) complex V inhibitor), 1 µmol/L of FCCP (mitochondrial uncoupler), or 2.5 µmol/L of antimycin A (ETC complex III inhibitor) combined with 0.5 µmol/L of rotenone (ETC complex I inhibitor) for 4 h. (**A**) Incomplete oxidation measured as acid-soluble metabolites (ASM). (**B**) Complete oxidation measured as trapped CO_2_. (**C**) Total lipid accumulation and (**D**) lipid distribution. Values are presented as the mean ± SEM in absolute values as nmol/mg of protein. Data are from three individual experiments, each with two biological replicates (*n* = 6). * Statistically significant in cPANC-1-exposed versus chPEC-exposed myotubes (*p* ≤ 0.05, paired *t*-test). CE, cholesteryl ester; DAG, diacylglycerol; FFA, free fatty acids; PL, phospholipid; TAG, triacylglycerol.

We previously showed that PANC-1-conditioned myotubes had reduced ability to oxidize energy substrates compared to non-conditioned myotubes [10]. In this study, we found no difference in incomplete fatty acid oxidation measured as ASM (Figure 5A) or basal complete fatty acid oxidation to CO_2_ (Figure 5B) in cPANC-1-exposed compared to chPEC-exposed myotubes. Mitochondrial dysfunction is a common feature of cancer cachexia, which occurs when the mitochondria work less efficiently than they should due to another disease or condition, such as cachexia [4] or aging [16]. We explored the mitochondrial responsiveness by using electron transport chain (ETC) inhibitors. Treatment with oligomycin or a combination of rotenone and antimycin A revealed that cPANC-1-exposed myotubes were less sensitive to the inhibitors; thus, the oxidation of oleic acid after inhibitor exposure was higher in cPANC-1-exposed cells compared to chPEC-exposed myotubes (Figure 5B). Further, total lipid accumulation from oleic acid and the level of free oleic acid (FFA) were higher in cPANC-1-exposed cells compared to chPEC-exposed cells (Figure 5C,D). Incorporation of oleic acid into free fatty acids (FFA), triacylglycerol (TAG), and cholesterol ester (CE) was also higher in cPANC-1-exposed myotubes compared to chPEC-exposed cells (Figure 5D).

**Figure 6:**
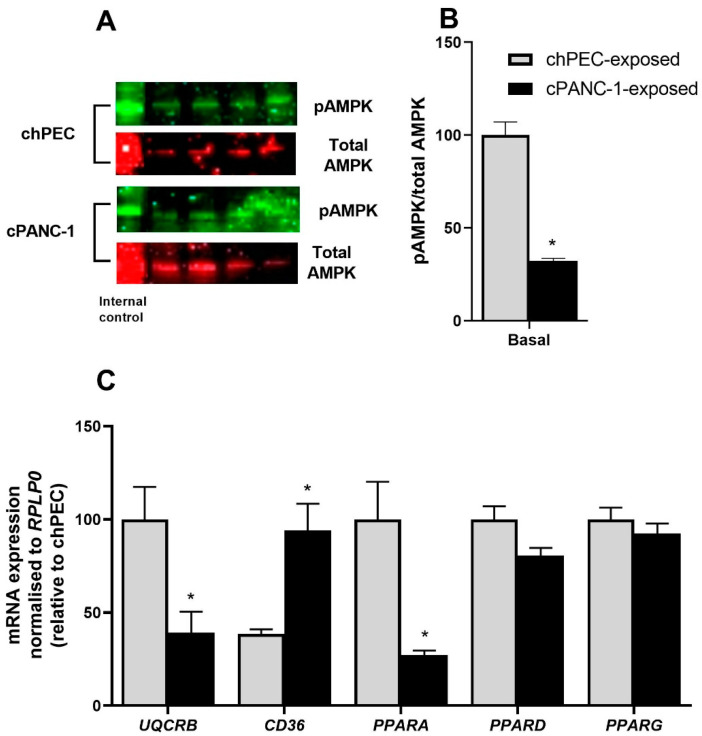
AMPK phosphorylation and gene expression in PANC-1- and hPEC-conditioned myotubes. In the last 4 days of differentiation, myotubes were conditioned with the medium from human primary pancreatic epithelial cells (chPEC exposed) or a pancreatic cancer cell line (cPANC-1 exposed). (**A**) Immunoblot showing AMPK phosphorylated on threonine 172 (pAMPK) and total AMPK. (**B**) Quantified immunoblots showing the pAMPK/total AMPK ratio adjusted to the internal control (HEK cells) and presented as a percentage of basal chPEC. (**C**) mRNA expression of ETC complex III (*UQCRB*), fatty acid transporter (*CD36*), and peroxisome proliferator-activated receptor alpha (*PPARA*), delta (*PPARD*) and gamma (*PPARG*). Data in (**B**,**C**) are from three individual experiments, each with two biological replicates, presented as the mean ± SEM (*n* = 6). * Statistically significant in cPANC-1-exposed versus chPEC-exposed myotubes (*p* ≤ 0.05, paired *t*-test).

The phosphorylation of AMPK at threonine 172, relative to total AMPK, was also markedly lower in cPANC-1-exposed myotubes compared to chPEC-exposed cells (Figure 6A,B). Lastly, mRNA expression of the fatty acid transporter *CD36* (fatty acid translocase, FAT) was higher, while mRNA expression of the ETC complex III (*UQCRB*) and the nuclear receptor peroxisome proliferator-activated receptor alpha (*PPARA*) was lower in cPANC-1-exposed myotubes compared to chPEC-exposed cells (Figure 6C).

### 3.5. Secretomes from hPECs and PANC-1 Cells Indicate Increased Secretion of Certain Cytokines, Growth Factors, and Growth-Factor-Binding Proteins Potentially Affecting Metabolism in Skeletal Muscle Cells

Secretomes from hPECs and PANC-1 cells were examined using quantitative label-free proteomics (data previously published [10]). Cytokines, growth factors, and growth-factor-binding proteins in the secretomes were classified using STRING software (Table 1). Most of these proteins were more abundant in PANC-1 medium compared to hPEC medium, including stem cell factor KIT ligand (KITLG), fibroblast growth factor 19 (FGF19), metallopeptidase inhibitor 1 (TIMP1), insulin-like growth factor 2 (IGF2), and insulin-like growth-factor-binding protein 2 (IGFBP2), while the amount of transforming growth factor beta-2 (TGFB2) reduced (Table 1).

## 4. Discussion

In this study, we found that myotubes exposed to a conditioned medium from PANC-1 cells had decreased protein content compared to myotubes exposed to hPEC medium. The PANC-1-medium-exposed myotubes also showed decreased overall leucine accumulation, increased overall leucine decay, and decreased gene expression of *MYH2*. Further, glucose uptake and oxidation decreased in cPANC-1-exposed cells. Moreover, we observed higher total cellular accumulation of oleic acid and a higher amount of FFA, TAG, and CE, supported by increased *CD36* gene expression, in cPANC-1-exposed myotubes compared to chPEC-exposed cells. Basal fatty acid oxidation was equal in chPEC-exposed and cPANC-1-exposed myotubes; however, ETC complex inhibitors had less impact on cPANC-1-exposed cells, and mRNA expression of the ETC-complex-III-related gene *UQCRB* reduced in cPANC-1-exposed myotubes.

Imbalance in protein metabolism due to hyperactive catabolism of muscle protein and decreased muscle protein mass is a common hallmark of cancer cachexia and is often already present at the time of diagnosis for many patients with pancreatic cancer [17]. When exploring the protein content in PANC-1-exposed myotubes, we found that the protein content decreased. Therefore, we further explored leucine accumulation and decay in cPANC-1-exposed myotubes. We found decreased accumulation and increased decay of radioactive leucine, suggesting an overall decrease in protein in these cells. This was also evident when comparing the protein amount from the two different cells, as the protein content was lower in cPANC-1-exposed compared to chPEC-exposed myotubes. In addition, the gene expression of *MYH2* was significantly lower in cPANC-1-exposed myotubes. *MYH2* is one of the three isoforms of myosin heavy chain, encoding a myosin heavy chain expressed in fast-twitch-type 2A fibers [18], the others being *MYH1*, expressed in fast-twitch-type 2X fibers, and *MYH7*, expressed in slow-twitch-type 1 fibers [19]. Grown in culture, human satellite cells mature into myotubes that express all three isoforms [20]. Mice studies have uncovered selective atrophy in type II fiber types during cachexia [21]. Muscle wasting in cancer cachexia is linked to increased NFκB activation in muscle [22]; however, we observed no difference in *NFκB* gene expression between our conditioned myotubes. The serine-threonine kinase mTOR is ubiquitously expressed and part of two different protein complexes named mTORC1 and mTORC2 [23]. The role of mTOR in cancer cachexia seems rather two-faced. On the one hand, cachexic muscle has reduced gene expression of mTOR complex 1 (*mTORC1*), which results in decreased protein and lipid synthesis; on the other hand, inhibiting *mTORC1* protects against cachexia by upregulating autophagy and inhibiting pro-cachectic factors [23]. We observed no significant difference in *MTOR* mRNA expression between cPANC-1-exposed and chPEC-exposed myotubes.

Further, we found that cPANC-1-exposed myotubes had lower uptake and oxidation of glucose compared to chPEC-exposed myotubes, whereas insulin-induced glycogen synthesis was not different between the two conditions. Liu et al. showed that the diabetes typically found in patients with pancreatic cancer is characterized by profound peripheral insulin resistance, which impairs skeletal muscle glycogen synthesis and storage, and is also associated with a post-insulin resistance defect and no difference in *SLC2A4* gene expression [24]. The insulin receptor is not thought to be affected in pancreatic-cancer-related diabetes, which suggests that the defect might be further down the cascade toward glucose uptake and glycogen synthesis.

In our experiments, total lipid accumulation was higher in cPANC-1-exposed compared to chPEC-exposed myotubes and significantly more oleic acid was found as FFA or incorporated into TAG and CE, accompanied by increased gene expression of *CD36*. Tumor-bearing animals have shown to have a 60% increase in FAT in skeletal muscle [25]. Disruption of normal mitochondrial activity, leading to mitochondrial dysfunction, is associated with lipid metabolic disorders. However, increased lipid accumulation (i.e., TAG accumulation) can also lead to mitochondrial dysfunction [26]. The accumulation of cytosolic fatty acids will, in turn, increase the production of reactive oxygen species, which then leads to decreased mitochondrial biogenesis, increased mutation rates, and aging-like impaired mitochondrial function [27]. When comparing fatty acid oxidation in the conditioned myotubes, we observed decreased effects of mitochondrial inhibitors in cPANC-1-exposed cells. These are similar findings as seen in the mitochondria of aging cells [16]. Previous work comparing satellite cells from old versus young mice found that the muscle cells from young mice showed a decrease in basal respiration after addition of oligomycin, while the cells from old mice were less responsive, indicating that these cells already were respiring at minimal capacity [28]. However, the cells from old mice remained responsive to FCCP, though to a lesser extent than the cells from young mice [28]. This is similar to our results and indicates aging-like impaired mitochondrial function in myotubes after cPANC-1 exposure. Additionally, mitochondrial dysfunction in subjects with lipid storage diseases was associated with significant loss of activity in ETC complex I-IV compared to the control [29]. Studies on rat muscle cells found that only small changes in complex III activity (approximately 5% inhibition) was sufficient to change respiration [30]. Similar to these findings, we found a lower mRNA level of ETC complex III, *UQCRB*, in our cPANC-1-exposed myotubes compared to chPEC-exposed cells.

AMPK phosphorylation related to total AMPK was significantly lower in cPANC-1-exposed myotubes. As a major cellular energy sensor, AMPK acts as a moderator of skeletal muscle lipid metabolism [31]. Activation of AMPK through exercise is found to be beneficial as it increases glucose uptake independent of insulin [31]. AMPK also increases oxidative phosphorylation in skeletal muscle through transcriptional regulation [31]. The phosphorylation of AMPKα is inversely correlated with lipid accumulation in murine skeletal muscle C2C12 cells [31]. This is in line with the increased accumulation of lipids found in our cell model after cPANC-1 exposure, also linked to the typically increased lipid accumulation seen in cells with mitochondrial dysfunction [27].

Investigating relevant genes, we found a significant decrease in *PPARA* gene expression in cPANC-1-exposed compared to chPEC-exposed myotubes. PPARs are important metabolic regulators in the body, with different expression patterns across metabolic tissues. They are also involved in tumor development, though expression levels vary between different tumor types and stages of cancer [32]. Although PPARα agonists have been reported to show antitumor effects in colon carcinogenesis, it is still disputed whether PPARα represses or promotes cancer [32]. In patients with cachexia due to chronic obstructive pulmonary disease (COPD), *PPARA* mRNA expression decreased compared to patients with non-cachectic COPD [33]. PPARα may play a role in glucose usage in aged muscle, and decreased muscle glycogen concentrations have been detected in aged PPARα-deficient mice [34]. This supports our findings on mitochondria and glucose handling, implying an interference of the cancer cell secretome on myotube energy metabolism, causing decreased mitochondrial function and thereby increased lipid accumulation.

Previously, we showed the different metabolic profiles of hPECs and PANC-1 cells, where PANC-1 cells oxidized more glucose and less oleic acid compared to hPECs [10]. In addition, PANC-1 cells secreted a higher amount of proteins compared to hPECs [10]. Secreted substances from pancreatic cancer cells have been proposed to alter metabolism in skeletal muscle and induce peripheral insulin resistance and cachexia in skeletal muscle [7]. When comparing the proteins secreted from PANC-1 cells and hPECs, we found a higher abundance of some growth factors and growth-factor-binding proteins in the conditioned medium from PANC-1 cells compared to that from hPECs. These included the stem cell factor KITLG, often found to be upregulated in several types of cancers [35]; fibroblast growth factor 19 (FGF19), whose upregulation is linked to hepatocellular carcinoma and unfavorable outcomes [36]; and tissue inhibitor of metalloproteinases-1 (TIMP1), which is typically upregulated in patients with PDAC [37]. TIMP1 is associated with a poor clinical outcome for cancers [38] and is suggested as a biomarker for cachexia [39]. In addition, IGF2 and IGFBP2 were more abundant in the secretome of PANC-1 cells compared to hPECs. These are tumor growth promotors and, therefore, often increased in different tumors [40]. Increased IGF2 has also been found to lead to activation of Akt [41]. This could potentially explain the higher basal level of Akt phosphorylation seen in our cPANC-1-exposed compared to chPEC-exposed myotubes. Previously, both increased and decreased Akt activity was observed during cancer cachexia [23].

Several of the typical circulating factors during cachexia, such as IL-1, IL-6, and TNFα [4], were not found in the secretome from PANC-1 cells and hPECs. However, in vivo, these factors or cytokines might come via the tumor cells’ interaction with immune cells or other cells, which was missing in our in vitro experiments only using pancreatic cells and skeletal muscle cells. We also need to consider that there might be other factors in conditioned media influencing the myotubes. To mention one, we previously showed that glucose levels were significantly lower in PANC-1 medium compared to hPEC medium, while lactate levels significantly increased [10]. Although glucose levels were adjusted to be equal in the conditioned media in this study, lactate levels were not corrected.

Our data support the suggestion that cancer cells release substances that impact metabolism in skeletal muscle cells. In addition, an overall reduction in protein content, decreased gene expression of *MYH2,* decreased leucine accumulation, and increased leucine decay found in myotubes conditioned with PANC-1 medium indicate that the cancer cell secretome may affect the muscle protein content with similarities to the cachexia often seen in cancer. Our results also might indicate mitochondrial dysfunction in PANC-1-conditioned myotubes, an important component in the wasting disorder often preceding atrophy development. A limitation of our study is that only a few selected markers associated with cachexia were examined and that the activity of several signaling pathways involved in muscle atrophy (Li et al., 2021) was not examined [42]. Another limitation is that we are solemnly studying the effects of conditioning primary human skeletal muscle cells with media from pancreatic cells using an in vitro system, as described before. Cachexia is a complicated process involving different cells and organs, and in vivo, there is a much more extensive cross-talk network [4]. In addition, our findings are results from one pancreatic cancer cell line, and the implications of conditioning primary human myotubes with media from other pancreatic cell lines might give different results.

## 5. Conclusions

Our data showed that substances released from a pancreatic cancer cell line can induce changes in energy metabolism and protein turnover in primary human myotubes. Changes in glucose and oleic acid metabolism, as well as leucine turnover, are affected in a manner resembling cachexia. Our research contributes to pancreatic cancer research; however, further studies are needed to elucidate the mechanistic pathways leading to cancer-induced changes in skeletal muscle metabolism.

## Figures and Tables

**Table 1:** Cytokines, growth factors, and growth-factor-binding proteins differently secreted by PANC-1 cells and hPECs.

	Protein	log10 Fold-Change PANC-1 Cells vs. hPECs
VGF	Neurosecretory protein VGF	3.175
KITLG	Kit ligand	2.381
AREG	Amphiregulin	1.810
LTBP3	Latent-transforming growth factor beta-binding protein 3	1.779
IGFBP2	Insulin-like growth-factor-binding protein 2	1.694
LTBP4	Latent-transforming growth factor beta-binding protein 4	1.594
FGF19	Fibroblast growth factor 19	1.510
TIMP1	Metalloproteinase inhibitor 1	1.392
GMFB	Glia maturation factor beta	1.338
BMP1	Bone morphogenetic protein 1	1.362
MIF	Macrophage migration inhibitory factor	0.887
IGF2	Insulin-like growth factor II	0.885
TGFB2	Transforming growth factor beta-2	−1.110

Color coding: growth factors (annotated keywords in STRING), growth-factor-binding proteins (annotated keywords in STRING), and cytokines.

## Data Availability

Mass-spectrometry-based proteomics have previously been used to study global protein secretion from the two cell types. The full data set has been deposited to the ProteomeXchange Consortium via the PRIDE partner repository with dataset identifier PXD017613.

## Supplementary Materials

The following supporting information can be downloaded at: https://www.mdpi.com/article/10.3390/metabo12111095/s1, Table S1: Description of primers.

## References

[1] Adamska A., Domenichini A., Falasca M. (2017). Pancreatic Ductal Adenocarcinoma: Current and Evolving Therapies. Int. J. Mol. Sci..

[2] Hanahan D., Weinberg R.A. (2011). Hallmarks of cancer: The next generation. Cell.

[3] Sandri M. (2016). Protein breakdown in cancer cachexia. Semin. Cell Dev. Biol..

[4] Siddiqui J.A., Pothuraju R., Jain M., Batra S.K., Nasser M.W. (2020). Advances in cancer cachexia: Intersection between affected organs, mediators, and pharmacological interventions. Biochim. Biophys. Acta-Rev. Cancer.

[5] Kunzke T., Buck A., Prade V.M., Feuchtinger A., Prokopchuk O., Martignoni M.E., Heisz S., Hauner H., Janssen K.P., Walch A. (2020). Derangements of amino acids in cachectic skeletal muscle are caused by mitochondrial dysfunction. J. Cachexia Sarcopenia Muscle.

[6] Holloszy J.O. (2008). Skeletal muscle “mitochondrial deficiency” does not mediate insulin resistance. Am. J. Clin. Nutr..

[7] Permert J., Adrian T.E., Jacobsson P., Jorfelt L., Fruin A.B., Larsson J. (1993). Is profound peripheral insulin resistance in patients with pancreatic cancer caused by a tumor-associated factor?. Am. J. Surg..

[8] Röhrig F., Schulze A. (2016). The multifaceted roles of fatty acid synthesis in cancer. Nat. Rev. Cancer.

[9] Wang F., Herrington M., Larsson J., Permert J. (2003). The relationship between diabetes and pancreatic cancer. Mol. Cancer.

[10] Krapf S.A., Lund J., Lundkvist M., Dale M.G., Nyman T.A., Thoresen G.H., Kase E.T. (2020). Pancreatic cancer cells show lower oleic acid oxidation and their conditioned medium inhibits oleic acid oxidation in human myotubes. Pancreatology.

[11] Wensaas A.J., Rustan A.C., Lovstedt K., Kull B., Wikstrom S., Drevon C.A., Hallen S. (2007). Cell-based multiwell assays for the detection of substrate accumulation and oxidation. J. Lipid Res..

[12] Bradford M.M. (1976). A rapid and sensitive method for the quantitation of microgram quantities of protein utilizing the principle of protein-dye binding. Anal. Biochem..

[13] Gaster M., Rustan A.C., Aas V., Beck-Nielsen H. (2004). Reduced Lipid Oxidation in Skeletal Muscle From Type 2 Diabetic Subjects May Be of Genetic Origin. Diabetes.

[14] Bakke S.S., Moro C., Nikolić N., Hessvik N.P., Badin P.-M., Lauvhaug L., Fredriksson K., Hesselink M.K., Boekschoten M.V., Kersten S. (2012). Palmitic acid follows a different metabolic pathway than oleic acid in human skeletal muscle cells; lower lipolysis rate despite an increased level of adipose triglyceride lipase. Biochim. Biophys. Acta-Mol. Cell Biol. Lipids.

[15] Hessvik N.P., Bakke S.S., Fredriksson K., Boekschoten M.V., Fjorkenstad A., Koster G., Hesselink M.K., Kersten S., Kase E.T., Rustan A.C. (2010). Metabolic switching of human myotubes is improved by n-3 fatty acids. J. Lipid Res..

[16] Srivastava S. (2017). The Mitochondrial Basis of Aging and Age-Related Disorders. Genes.

[17] Poulia K.A., Sarantis P., Antoniadou D., Koustas E., Papadimitropoulou A., Papavassiliou A.G., Karamouzis M.V. (2020). Pancreatic Cancer and Cachexia-Metabolic Mechanisms and Novel Insights. Nutrients.

[18] Madigan N.N., Polzin M.J., Cui G., Liewluck T., Alsharabati M.H., Klein C.J., Windebank A.J., Mer G., Milone M. (2021). Filamentous tangles with nemaline rods in MYH2 myopathy: A novel phenotype. Acta Neuropathol. Commun..

[19] Schiaffino S., Reggiani C. (2011). Fiber Types in Mammalian Skeletal Muscles. Physiol. Rev..

[20] Nikolić N., Bakke S.S., Kase E.T., Rudberg I., Flo Halle I., Rustan A.C., Thoresen G.H., Aas V. (2012). Electrical pulse stimulation of cultured human skeletal muscle cells as an in vitro model of exercise. PLoS ONE.

[21] Acharyya S., Butchbach M.E.R., Sahenk Z., Wang H., Saji M., Carathers M., Ringel M.D., Skipworth R.J.E., Fearon K.C.H., Hollingsworth M.A. (2005). Dystrophin glycoprotein complex dysfunction: A regulatory link between muscular dystrophy and cancer cachexia. Cancer Cell.

[22] Chen M.-C., Hsu W.-L., Hwang P.-A., Chen Y.-L., Chou T.-C. (2016). Combined administration of fucoidan ameliorates tumor and chemotherapy-induced skeletal muscle atrophy in bladder cancer-bearing mice. Oncotarget.

[23] Duval A.P., Jeanneret C., Santoro T., Dormond O. (2018). mTOR and Tumor Cachexia. Int. J. Mol. Sci..

[24] Liu J., Knezetic J.A., Strömmer L., Permert J., Larsson J., Adrian T.E. (2000). The Intracellular Mechanism of Insulin Resistance in Pancreatic Cancer Patients. J. Clin. Endocrinol. Metab..

[25] Fuster G., Busquets S.l., Ametller E., Olivan M., Almendro V., Fontes de Oliveira C.C., Figueras M., López-Soriano F.J., Argilés J.M. (2007). Are Peroxisome Proliferator-Activated Receptors Involved in Skeletal Muscle Wasting during Experimental Cancer Cachexia? Role of β_2_-Adrenergic Agonists. Cancer Res..

[26] Guo C., Sun L., Chen X., Zhang D. (2013). Oxidative stress, mitochondrial damage and neurodegenerative diseases. Neural Regen. Res..

[27] Jana B.A., Chintamaneni P.K., Krishnamurthy P.T., Wadhwani A., Mohankumar S.K. (2019). Cytosolic lipid excess-induced mitochondrial dysfunction is the cause or effect of high fat diet-induced skeletal muscle insulin resistance: A molecular insight. Mol. Biol. Rep..

[28] Pala F., Di Girolamo D., Mella S., Yennek S., Chatre L., Ricchetti M., Tajbakhsh S. (2018). Distinct metabolic states govern skeletal muscle stem cell fates during prenatal and postnatal myogenesis. J. Cell Sci..

[29] Debashree B., Kumar M., Keshava Prasad T.S., Natarajan A., Christopher R., Nalini A., Bindu P.S., Gayathri N., Srinivas Bharath M.M. (2018). Mitochondrial dysfunction in human skeletal muscle biopsies of lipid storage disorder. J. Neurochem..

[30] Taylor R.W., Birch-Machin M.A., Bartlett K., Lowerson S.A., Turnbull D.M. (1994). The control of mitochondrial oxidations by complex III in rat muscle and liver mitochondria. Implications for our understanding of mitochondrial cytopathies in man. J. Biol. Chem..

[31] Jørgensen S.B., Richter E.A., Wojtaszewski J.F.P. (2006). Role of AMPK in skeletal muscle metabolic regulation and adaptation in relation to exercise. J. Physiol..

[32] Takada I., Makishima M. (2020). Peroxisome proliferator-activated receptor agonists and antagonists: A patent review (2014-present). Expert Opin. Ther. Pat..

[33] Remels A.H., Schrauwen P., Broekhuizen R., Willems J., Kersten S., Gosker H.R., Schols A.M. (2007). Peroxisome proliferator-activated receptor expression is reduced in skeletal muscle in COPD. Eur. Respir. J..

[34] Atherton H.J., Gulston M.K., Bailey N.J., Cheng K.K., Zhang W., Clarke K., Griffin J.L. (2009). Metabolomics of the interaction between PPAR-alpha and age in the PPAR-alpha-null mouse. Mol. Syst. Biol..

[35] Yang S., Li W.-S., Dong F., Sun H.-M., Wu B., Tan J., Zou W.-J., Zhou D.-S. (2014). KITLG is a novel target of miR-34c that is associated with the inhibition of growth and invasion in colorectal cancer cells. J. Cell. Mol. Med..

[36] Miura S., Mitsuhashi N., Shimizu H., Kimura F., Yoshidome H., Otsuka M., Kato A., Shida T., Okamura D., Miyazaki M. (2012). Fibroblast growth factor 19 expression correlates with tumor progression and poorer prognosis of hepatocellular carcinoma. BMC Cancer.

[37] Pan S., Chen R., Crispin D.A., May D., Stevens T., McIntosh M.W., Bronner M.P., Ziogas A., Anton-Culver H., Brentnall T.A. (2011). Protein alterations associated with pancreatic cancer and chronic pancreatitis found in human plasma using global quantitative proteomics profiling. J. Proteome Res..

[38] Seubert B., Grünwald B., Kobuch J., Cui H., Schelter F., Schaten S., Siveke J.T., Lim N.H., Nagase H., Simonavicius N. (2015). Tissue inhibitor of metalloproteinases (TIMP)-1 creates a premetastatic niche in the liver through SDF-1/CXCR4-dependent neutrophil recruitment in mice. Hepatology.

[39] Prokopchuk O., Grünwald B., Nitsche U., Jäger C., Prokopchuk O.L., Schubert E.C., Friess H., Martignoni M.E., Krüger A. (2018). Elevated systemic levels of the matrix metalloproteinase inhibitor TIMP-1 correlate with clinical markers of cachexia in patients with chronic pancreatitis and pancreatic cancer. BMC Cancer.

[40] Bergman D., Halje M., Nordin M., Engström W. (2013). Insulin-Like Growth Factor 2 in Development and Disease: A Mini-Review. Gerontology.

[41] Barton-Davis E.R., Shoturma D.I., Sweeney H.L. (1999). Contribution of satellite cells to IGF-I induced hypertrophy of skeletal muscle. Acta Physiol. Scand..

[42] Li Y., Jin H., Chen Y., Huang T., Mi Y., Zou Z. (2021). Cancer cachexia: Molecular mechanism and pharmacological management. Biochem. J..

