# Supplementary material for: Pancreatic Cancer Cell-Conditioned, Human-Derived Primary Myotubes Display Increased Leucine Turnover, Increased Lipid Accumulation, and Reduced Glucose Uptake
Source: Metabolites. 2022 Nov 10;12(11):1095. doi: 10.3390/metabo12111095 (PMC9696174; doi:10.3390/metabo12111095)
Supplement: Supplementary file 1 [file metabolites-12-01095-s001.zip › metabolites-1996254-supplementary.pdf]

Supplementary Table S1. Description of primers.

Gene	Acc.no.	Forward sequence	Reverse sequence
CD36	L06850	AGTCACTGCGACATGATTAATGGT	CTGCAATACCTGGCTTTTCTCAA
GAPDH	NM_002046	TGCACCACCACCTGCTTAGC	GGCATGGACTGTGGTCATGAG
SLC2A4	M20747	ACCCTGGTCCTTGCTGTGTT	ACCCCAATGTTGTACCCAAACT
MYH2	NM_005963	CCAGACTGTGTCTGCTCTCTTCAG	CAGGACAAGCTCATGCTCCAT
MTOR	NM_001386500	CCGAGCGACGAGAGATCATC	ACTTGAGCCAGGTTCTCATGTCT
MYOD1	BC064493	GCGCCCAAAAGATTGAACTTA	CCGCCTCTCCTACCTCAAGA
MAPK8	NM_001278547	CTGGTCAGCAGGGTGTCACA	TCGCAGAGGGAGAAAAGCAA
NFκB	NM001165412.1	ACGAGCTCCGAGACAGTGACA	GAGACTCGGTAAAGCTGAGTTTGC
PPARA	L02932	TCCACCTGCAGAGCAACCA	CCGGAGGTCTGCCATTTTT
PPARD	BC002715	AGCATCCTCACCGGCAAA	ATGTCTCGATGTCGATGTCGTGGATCAC
PPARG	L40904	AGCCTGCGAAAGCCTTTTG	ATTCCAGTGCATTGAACTTCACA
RPLP0	M17885	CCATTCTATCATCAACGGGTACAA	AGCAAGTGGGAAGGTGTAATCC
UQCRB	NM_006294.4	AAGGTCCAAGGTCTCCTCTCTTC	CATCTCCAGCAGGTACTTCACTCA

Primers were designed using Primer Express® (Applied Biosystems). *CD36*, cluster of differentiation 36; *GAPDH*, glyceraldehyde 3-phosphate dehydrogenase; SLC2A4, solute carrier family 2 member 4; *MYH2*, myosin heavy chain 2; *MTOR*, mechanistic target of rapamycin kinase; *MYOD1*, myoblast determination protein 1;MAPK8, mitogen-activated protein kinase 8; *NFκB*, nuclear factor kappa B; *PPARA/D/G*, peroxisome proliferator-activated receptor alpha/delta/gamma; *RPLP0*, ribosomal protein lateral stalk subunit P0; *UQCRB*, ubiquinol-cytochrome c reductase binding protein.
